# Patient Monitoring Alarms in an Intensive Care Unit: Observational Study With Do-It-Yourself Instructions

**DOI:** 10.2196/26494

**Published:** 2021-05-28

**Authors:** Akira-Sebastian Poncette, Maximilian Markus Wunderlich, Claudia Spies, Patrick Heeren, Gerald Vorderwülbecke, Eduardo Salgado, Marc Kastrup, Markus A Feufel, Felix Balzer

**Affiliations:** 1 Department of Anesthesiology and Intensive Care Medicine Charité – Universitätsmedizin Berlin Corporate Member of Freie Universität Berlin, Humboldt-Universität zu Berlin, and Berlin Institute of Health Berlin Germany; 2 Institute of Medical Informatics Charité – Universitätsmedizin Berlin Corporate Member of Freie Universität Berlin, Humboldt-Universität zu Berlin, and Berlin Institute of Health Berlin Germany; 3 Department of Psychology and Ergonomics (IPA), Division of Ergonomics Technische Universität Berlin Berlin Germany

**Keywords:** digital health, patient monitoring, intensive care unit, technological innovation, data science, alarm fatigue, alarm management, patient safety, ICU, alarm system, alarm system quality, medical devices, clinical alarms

## Abstract

**Background:**

As one of the most essential technical components of the intensive care unit (ICU), continuous monitoring of patients’ vital parameters has significantly improved patient safety by alerting staff through an alarm when a parameter deviates from the normal range. However, the vast number of alarms regularly overwhelms staff and may induce alarm fatigue, a condition recently exacerbated by COVID-19 and potentially endangering patients.

**Objective:**

This study focused on providing a complete and repeatable analysis of the alarm data of an ICU’s patient monitoring system. We aimed to develop do-it-yourself (DIY) instructions for technically versed ICU staff to analyze their monitoring data themselves, which is an essential element for developing efficient and effective alarm optimization strategies.

**Methods:**

This observational study was conducted using alarm log data extracted from the patient monitoring system of a 21-bed surgical ICU in 2019. DIY instructions were iteratively developed in informal interdisciplinary team meetings. The data analysis was grounded in a framework consisting of 5 dimensions, each with specific metrics: alarm load (eg, alarms per bed per day, alarm flood conditions, alarm per device and per criticality), avoidable alarms, (eg, the number of technical alarms), responsiveness and alarm handling (eg alarm duration), sensing (eg, usage of the alarm pause function), and exposure (eg, alarms per room type). Results were visualized using the R package ggplot2 to provide detailed insights into the ICU’s alarm situation.

**Results:**

We developed 6 DIY instructions that should be followed iteratively step by step. Alarm load metrics should be (re)defined before alarm log data are collected and analyzed. Intuitive visualizations of the alarm metrics should be created next and presented to staff in order to help identify patterns in the alarm data for designing and implementing effective alarm management interventions. We provide the script we used for the data preparation and an R-Markdown file to create comprehensive alarm reports. The alarm load in the respective ICU was quantified by 152.5 (SD 42.2) alarms per bed per day on average and alarm flood conditions with, on average, 69.55 (SD 31.12) per day that both occurred mostly in the morning shifts. Most alarms were issued by the ventilator, invasive blood pressure device, and electrocardiogram (ie, high and low blood pressure, high respiratory rate, low heart rate). The exposure to alarms per bed per day was higher in single rooms (26%, mean 172.9/137.2 alarms per day per bed).

**Conclusions:**

Analyzing ICU alarm log data provides valuable insights into the current alarm situation. Our results call for alarm management interventions that effectively reduce the number of alarms in order to ensure patient safety and ICU staff’s work satisfaction. We hope our DIY instructions encourage others to follow suit in analyzing and publishing their ICU alarm data.

## Introduction

### Background

In intensive care units (ICUs), monitoring of patients’ physiologic parameters has significantly improved patient safety by alerting ICU staff through a visual or audible alarm [1] when a parameter deviates from the preset range (eg, apnea, sensor detachment). However, as one of the most digitized health care areas with a rising number of novel devices with their own alarms, the sheer number of alarms regularly overwhelms ICU staff. Some studies document more than 700 alarms per patient per day on average [2].

Being exposed to so many alarms can leave ICU staff alarm fatigued, a condition characterized by a desensitization to alarms, which can make ICU staff react inadequately (eg, responding with delay, turning down the alarm volume, turning alarms off) [3,4]. Due to the COVID-19 pandemic, this condition has been further exacerbated (eg, through the utilization of anesthesia ventilators in the ICU) [5]. Excessive alarms not only induce stress and distraction in ICU staff [6,7] but also directly impair patient recovery [8]. Ultimately, it can threaten patients’ lives when ICU staff misses alarms or responds with delay. This is shown by the Joint Commission's sentinel event database, which lists 98 incidents between 2009 and 2012 that were related to alarms, of which 80 resulted in a patient’s death [9]. Reporting to the database is voluntary, which likely makes this a conservative estimate. For 2020, the ECRI Institute listed an alarm-related hazard among their Top 10 Health Technology Hazards [10]. While in the United States, the Joint Commission declared it a national patient safety goal to reduce the harm associated with clinical alarm systems from 2014 onwards [11]; there is no such official endeavor in Germany.

One way to reduce harm associated with clinical alarms is alarm management, which aims to reduce the number of unnecessary alarms (that is, false, nonactionable, and avoidable technical alarms [12]) with the assumption that this reduces the overall number of alarms and thereby alleviates the staff’s alarm fatigue. Traditional alarm management approaches that have been proven to reduce the overall number of alarms include the recommendation to mute alarms while examining a patient [13], introduce a delay between measuring and alarming [14], use individual thresholds for each patient instead of the monitoring device’s default [15], turn off arrhythmia alarms that are not life threatening, and change electrocardiogram (ECG) leads on a daily basis [16].

To be most effective, alarm management should be adjusted to the specific conditions of each ICU [12,17]. A thorough analysis of the sociotechnical system of the ICU is necessary to sufficiently customize respective interventions. These efforts include the analysis of the alarm log data (eg, when an alarm occurred and by which device) [12,18]. Currently there is no software solution commercially available that addresses analysis of patient monitoring data.

### Aim

Our aim is to develop do-it-yourself (DIY) instructions targeted at technically versed ICU staff (physicians and nurses) for self-analysis of patient monitoring alarm data, including an illustrative, complete, and repeatable analysis of device alarm data of an ICU’s patient monitoring system. The application of the DIY instructions should help their users to identify patterns and trends in the alarm data and enable them to generate ideas on how the overall alarm frequency (and subsequently alarm fatigue) might be reduced.

## Methods

### Ethics Approval

The ethical approval for this study was granted by the Ethics Commission of the Charité – Universitätsmedizin Berlin (EA1/127/18).

### Setting and Design

We conducted the study in a surgical ICU of a German university hospital. The unit consists of 21 beds in 15 rooms in which mainly patients after abdominal or neurosurgical operations are treated. The patient monitoring and alarm system used at the time of the study was the Philips IntelliVue patient monitoring system (MX800 software version M.00.03; MMS X2 software version H.15.41-M.00.04; Philips, Amsterdam, Netherlands) with bedside monitors, 3 client monitors summarizing 2-3 rooms, a central station (software version B), and 2 large hallway monitors displaying all 21 patients. Standard monitoring included oxygen saturation (SpO_2_), heart rate, invasive (IBP) or noninvasive blood pressure (NIBP), and temperature. Within the Clinical Alarm Capability Maturity Model by Welch et al [19], the ICU was in the first stage at the time of this study, described as having many nonactionable alarms for unknown reasons, approaching alarm management ad hoc and not having or not consulting data to support change [19]. Accordingly, there was no hospital-wide consensus on alarm management.

We used an observational study design, which included retrospective data analysis of the patient monitoring alarm data. The DIY instructions were iteratively developed in informal interdisciplinary team meetings within the research group between February 2019 and November 2020 and adapted by the lessons learned from our own data analysis.

### Data Collection and Deidentification

We manually collected clinical audit logs (which include the alarm data) 3 times during 2019 (in winter, summer, and autumn) via a USB stick from the central patient monitoring device in the ICU as previously described by others. The clinical audit log consists of the time, bed number, alarm type (ie, parameter, device, alarm criticality), and alarm handling (eg, threshold adjustments, use of the pause function). Each log file contains data from 31 days.

No actual patient-identifying data elements were collected. For further deidentification, dates were shifted into the future by a pseudorandom offset for all patients; the bed number was replaced by a pseudonym. Day and night rhythm, weekends, the season, and the bed characteristic (double room, single room) were not affected by this process. The deidentified raw data can be retrieved from an open data repository [20].

### Data Analysis Framework

#### Overview

We organized our data analysis in a framework based on suggestions by Hüske-Kraus et al [12], who introduced quality dimensions along with metrics of an alarm system. Each dimension summarizes multiple metrics (Table 1).

**Table 1:** Data analysis framework applied in this study in line with the quality dimensions introduced by Hüske-Kraus et al [12] and including metrics suggested by Hüske-Kraus et al [12] as well as metrics suggested by other sources for each dimension, wherever possible.

Quality dimension	Definition	Metrics used in this study
Alarm load	Metrics related to the number of alarms	Alarms per bed per day, frequency of individual alarms, alarms per device, alarms per criticality (red, yellow, and blue; ie, alarm at high criticality, alarm at medium criticality, and technical alarm at low criticality, respectively), average temporal distribution of alarms and alarm flood conditions (10 or more alarms occurring within 10 minutes) [18]
Avoidable alarms	False-positive alarms, nonactionable alarms, and technical alarms	Technical alarms per bed per day, technical alarms per device
Responsiveness and alarm handling	Alarm duration, response time, muting of alarms, and corrective actions	Duration of alarms
Sensing	The quality of the technical infrastructure, such as consumable, overmonitoring, and undermonitoring	Average usage of the alarm pause function per bed per day, proper pause-to-pause ratio [12], redundant monitoring of physiological parameters
Exposure	How alarms are distributed in the unit	Average alarm frequencies per room and per bed per room type, number of beds issuing more alarms than the average

#### Data Analysis

We cleaned and analyzed the data with R [21] in combination with the packages *dplyr* [22], *tidyr* [23], and *stringr* [24]. We used the package *lubridate* [25] for date and time information and the package *ggplot2* [11] for the visualizations. The log entries were structured in 4 columns: Time, Bedname, Action, Devicename. New variables (eg, the time an alarm was generated and its criticality) were extracted for each log entry from the information contained in the column Action. In total, the alarm logs contain data from 93 days.

#### Alarm Load

Visualizing the frequency of individual alarm parameters helps to identify “bad actors” — alarms that occur much more frequently than others [8] while investigating the number of alarms each medical device issues — can help to prioritize alarm management interventions. The metric “alarms per bed per day” alone is not necessarily an indicator of the alarm load on respective ICUs and should be accompanied with information such as the criticality of the alarms (red, yellow, blue), the frequency of individual alarms, the frequency of alarms per device, the number of alarm flood conditions, and a temporal perspective. To conduct device-related analyses, we assigned each alarm parameter to 1 out of 7 devices (ventilator, ECG, IBP, intracranial pressure, temperature, NIBP, and pulse oximetry [SpO_2_]). Technical alarms included alarms with a blue criticality and general monitoring device–related alarms (such as missing patient information, low batteries, or interrupted arterial blood pressure measurements). Devices, where the absolute cumulative frequency of alarms was less than 500 in the dataset were only included in the overall count of alarms (Multimedia Appendix 1).

To visualize the average distribution of alarms across 24 hours, we calculated the average number of alarms of each 1-minute bin between 12:00 am and 11:59 pm for the 3 devices issuing the most alarms and applied the scatterplot smoothing function of the R package ggplot2 [26].

Alarm flood conditions are described as situations in the ICU where multiple alarms are triggered within a short time frame. Metrics related to alarm flood conditions provide information that allow an additional perspective on the alarm load of an ICU and take an acute overload of ICU staff by alarms into account [18]. We grouped the data per bed and split each bed’s data into 10-minute bins starting from the date-time stamp of the first log entry. All bins containing 10 or more alarms were counted as an alarm flood, as previously described [18].

#### Avoidable Alarms

Avoidable alarms are defined as nonactionable (including false positive alarms) or technical [12]. Since the alarm log data lack information on whether an alarm was true or false or if it was followed by a therapeutic intervention, we cannot provide metrics such as the positive predictive value of alarms. However, most technical alarms, whether they were responded to with an intervention or not, are avoidable nonetheless [12]. Therefore, we report the average number of technical alarms per bed per day as well as the individual frequency of technical alarms.

#### Responsiveness and Alarm Handling

We visualize the median alarm duration per medical device in absolute seconds and over the course of 24 hours for the 3 devices issuing the most alarms. The duration of an alarm was defined as the time difference between the timestamp of the log entry of a generated alarm and that of a terminated alarm. We opted for this method, because only the “true” time of generated alarms is documented but not the “true” time of terminated alarms, where only the time of the log entry is provided.

#### Sensing

Using the pause function of the monitoring devices is argued to prevent unnecessary alarms [16,27]. In the investigated ICU, the alarm pause function suspends all alarms for up to 3 minutes or until it is actively terminated by the medical team. This helps prevent alarms that would be triggered when, for example, a patient is being shifted from one position to another during a physical examination. A responsible use of the pause function demands its active termination to avoid undermonitoring of the patient due to the suspended alarms for the remainder of the pause function [12] once the health care provider leaves the patient. Hence, an alarm pause can be considered a proper pause if its duration is shorter than the monitor's default pause duration. The metric “proper pause-to-pause ratio” indicates the ratio of proper pauses to all pauses [12]. We count pauses that were re-enabled within 3 minutes after their termination as one continuous pause, since the default length might not have been sufficient for the bedside procedure.

We consider an overmonitoring of patients to be indicated when parameters that monitor the same physiological event, but stem from 2 different medical devices, are routinely issuing alarms.

#### Exposure

The present ICU has 2 different room types: a single bedroom and a double bedroom. We aimed to find out whether a bed in a single bedroom produces more, less, or equally as many alarms as a bed in a double bedroom and whether this differs depending on the alarm criticality. In total, there are 12 beds in double bedrooms and 9 beds in single bedrooms. The average alarm frequencies per bed per room type were calculated by dividing the number of alarms per room type by 12 or by 9, respectively.

## Results

### Overview

The results section is clustered into 2 parts: First, we provide the DIY instructions for self-analysis of alarm data from the patient monitoring system; second, we present the results of the illustrative alarm data analysis conducted on data from our ICU.

### DIY Instructions

#### Step-by-Step Procedure

**Figure 1:**
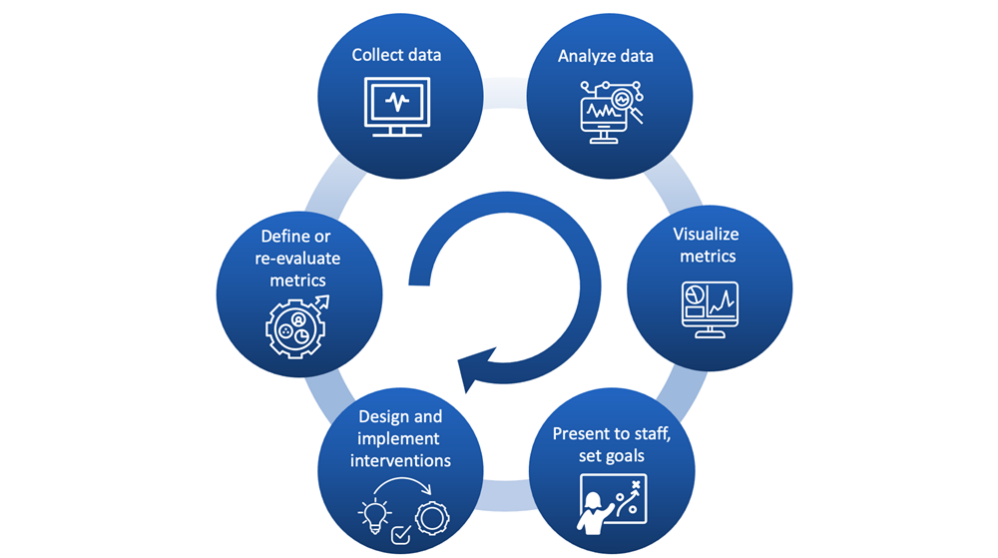
Feedback loop regarding do-it-yourself (DIY) instructions for self-analysis of patient monitoring alarm data in the intensive care unit.

The DIY instructions for self-analysis of the ICU’s patient monitoring alarm data consist of 6 steps: (1) define or re-evaluate metrics, (2) collect data, (3) analyze data, (4) visualize metrics, (5) present to staff and set goals together, and (6) design and implement interventions (see Figure 1).

#### Define or Re-Evaluate Metrics

In an interdisciplinary team consisting of ICU physicians and nurses, alarm data metrics should be defined. We recommend to initially include all metrics presented in the aforementioned data analysis framework in order to get an accurate and complete picture of the alarm situation of the respective unit. After the first iteration of the feedback loop, new metrics may be added or existing ones modified.

#### Collect Data

Regular collection of patient monitoring data is crucial to conduct reliable data analyses, especially if interventions are being conducted at the respective ICU. The monitoring central station at our ICU stores data for up to 90 days; hence, every 90 days, data have to be manually extracted from the system [28].

#### Analyze Data

We provide the fully annotated R scripts that we used to conduct the alarm data analysis to enable even beginners in R to do likewise. Further explanations can be found in the Results section and in the scripts [20].

#### Visualize Metrics, Present to Staff, and Set Goals

Visualizations should be summarized in a clear and intuitive format (eg, using a presentation program) and discussed with ICU staff. Together, realistic goals should be set for each parameter and possible interventions deduced. On a quarterly basis, this feedback loop should be started from the beginning. The R-Markdown file on GitHub can also be used to create comprehensive alarm reports including all metrics and visualizations reported in this paper [20].

#### Design and Implement Interventions

Potential interventions are deduced from the data analysis, the visualizations, and the interdisciplinary goal setting. ICU staff as the end users of the patient monitoring system should be actively involved in the intervention design and rotate regularly with new medical staff from the ICU to assess clinical relevance more reliably. Possible interventions could include adjusting the default alarm thresholds for the top 10 alarm parameters, focusing on customizing these parameters more frequently, introducing new ECG electrodes or skin preparation routines, or providing staff training (eg, on how to use the alarm pause function). Further interventions are elaborated elsewhere [29,30].

### Data Analysis

#### Alarm Load

**Figure 2:**
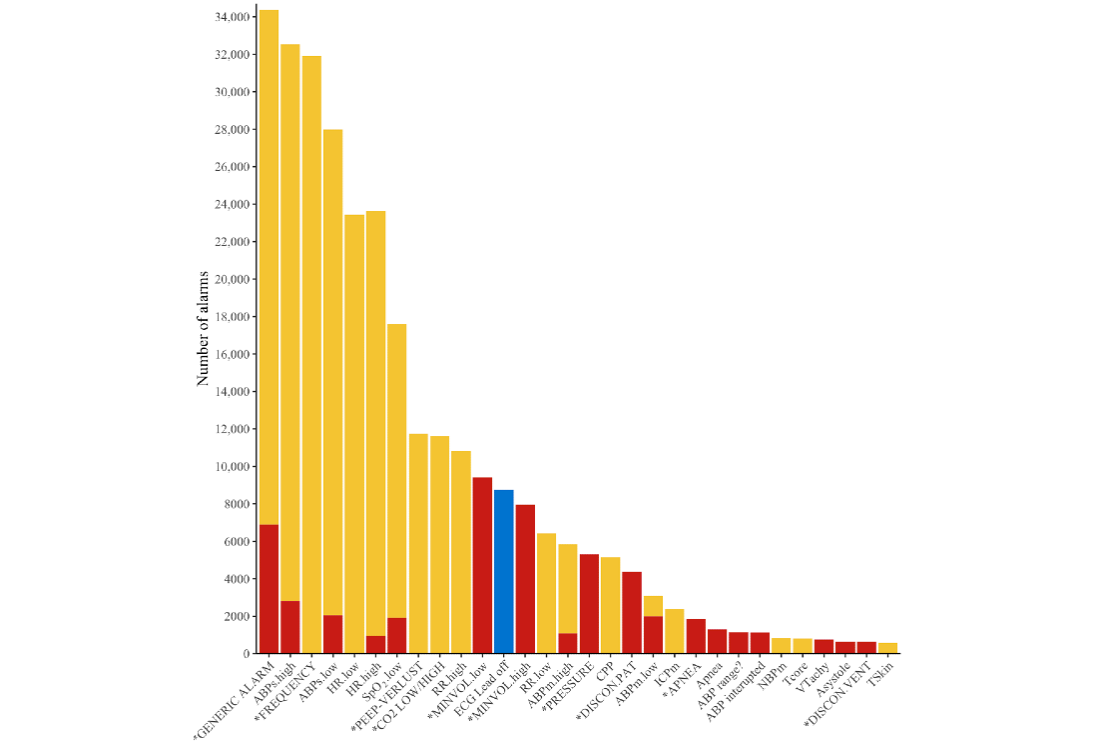
Frequency of individual alarm parameters within 93 days. The colors correspond to the alarm criticalities (red, yellow, and blue). *: ventilator arm; ABPs: systolic arterial blood pressure; ECG: electrocardiogram; FREQUENCY: ventilator alarm indicating that the upper respiratory rate threshold has been exceeded; HR: heart rate; RR: respiratory rate derived from the ECG (see Multimedia Appendix 2 for all abbreviations).

The analyzed alarm log data set contained, on average, 152.5 (SD 42.2) alarms per bed per day. Most alarms were type yellow (mean 120.3/152.5, SD 37.15 per day; 79%), followed by type red (mean 27.5/152.5, SD 9.37 per day; 18%). Few alarms were type blue (mean 4.6/152.5, SD 2.75 per day; 3%). The 5 most frequent alarms were a generic alarm of the ventilator, invasive systolic blood pressure (high and low), high respiratory rate issued by the ventilator, and low heart rate (see Figure 2).

**Figure 3:**
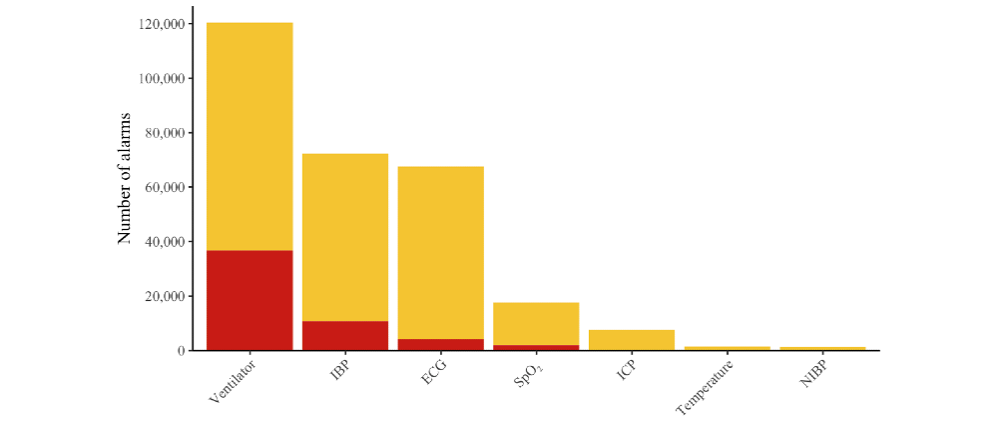
Alarms from medical devices within 93 days subdivided into the criticality levels (red, yellow). ECG: electrocardiogram; IBP: invasive blood pressure; ICP: intracranial pressure; NIBP: noninvasive blood pressure; SpO_2_: oxygen saturation.

After each alarm parameter was assigned to the corresponding medical device, it was evident that the ventilator generates the most alarms, followed by IBP and ECG (see Figure 3).

**Figure 4:**
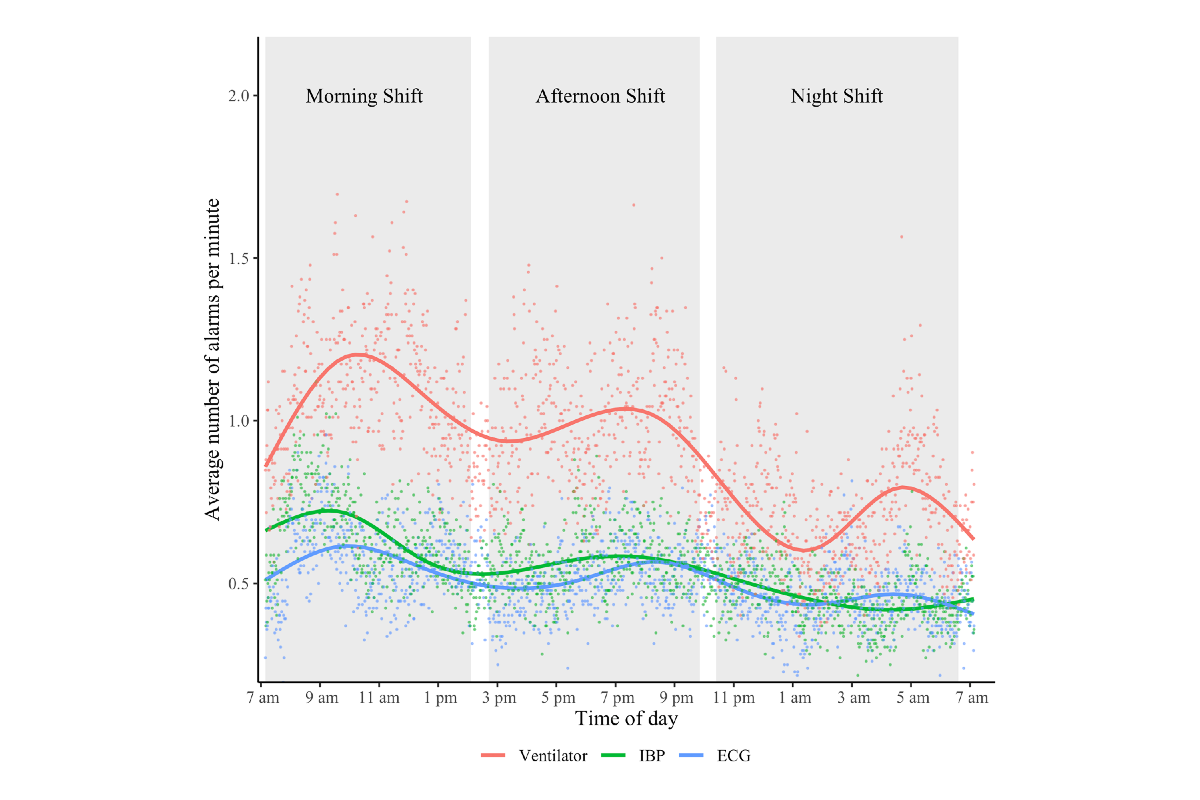
Average distribution of alarms across 24 hours. The white spaces between the grey bars (ie, shifts) visualize handover periods. Each dot shows the average alarm frequency of 1 minute for the specified device. The line for each device is calculated by ggplot2’s smoothing function and represents a generalized additive model of the distribution (with the formula y ~ s(x, bs = "cs"). It serves to aid in detecting trends in the data. ECG: electrocardiogram; IBP: invasive blood pressure.

When put into a temporal perspective, the average distribution of alarms across 24 hours for the 3 devices that issue the most alarms shows a downward trend, with most alarms being issued in the morning shift and fewest during the night (see Figure 4). The mean number of alarms per minute per medical device during the morning, afternoon, and night shifts were: 1.09 (SD 0.2), 1.0 (SD 0.18), and 0.71 (SD 0.17) for the ventilator; 0.65 (SD 0.13), 0.56 (SD 0.1), and 0.45 (SD 0.09) for IBP; and 0.57 (SD 0.12), 0.51 (SD 0.1), and 0.45 (SD 0.09) for ECG, respectively.

**Figure 5:**
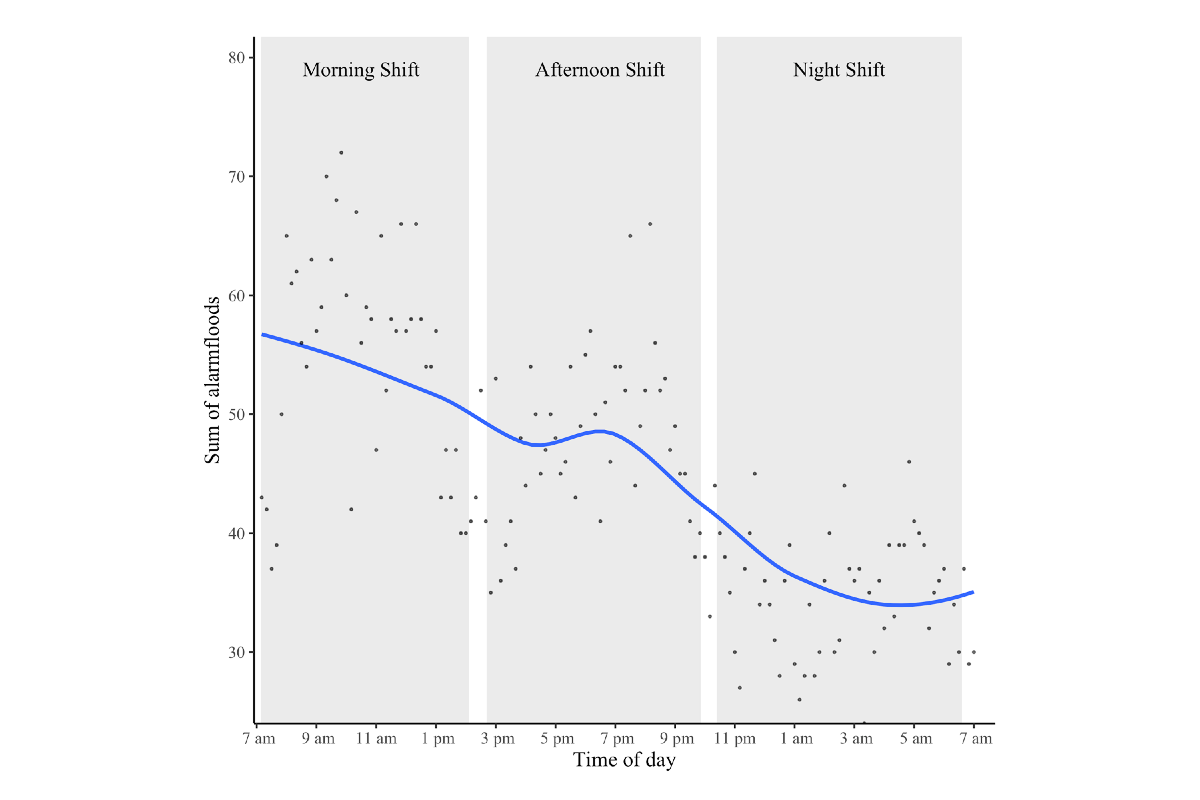
Temporal distribution of alarm flood conditions over 24 hours. Each dot indicates the sum of all alarm flood conditions that were initiated at the respective time of day in 10-minute intervals. For example, the first dot on the far left indicates that 43 alarm floods occurred between 7:10 and 7:20 AM across all days in the data. The blue line is a local regression, calculated by ggplot2’s smoothing function (formula: y ~ x). The white spaces between the grey bars (ie, shifts) visualize handover periods.

In total, 6468 alarm flood conditions occurred (mean 69.55, SD 31.0 per day; median 63; range 22-194), of which 5289 (82%) were comprised of between 10 and 20 alarms, 1012 (16%) between 20 and 40 alarms, and 159 (2%) between 40 and 100 alarms within 10-minute intervals. The temporal visualization over 24 hours shows a general downward trend with peaks in the morning and afternoon shifts (see Figure 5).

#### Avoidable Alarms

In total, 10,846 technical alarms (red, yellow, and blue) are documented. This equals 5.6 (SD 2.8) technical alarms per bed per day, on average. With 8746 alarms (all blue), the ECG produced the most technical alarms (ECG lead fallen off), followed by IBP (1342 red alarms) and alarms related to the module cable connection (167 blue alarms).

#### Responsiveness and Alarm Handling

The alarm duration showed a distribution that is strongly skewed to the right (for clinical alarms: mean 109.3, SD 6109.15 seconds; median 8 seconds; range 0-2,291,314 seconds; for technical alarms: mean 221.5, SD 4,898 seconds; median 7 seconds; range 0-403,440 seconds), which is why we used the median as the measure of the center for further analyses and plots. Additionally, because some durations were calculated to be unrealistically high (in some instances, multiple days), we treat all durations longer than 8 hours (approximately the length of one shift) as outliers and do not include them in the analyses.

**Figure 6:**
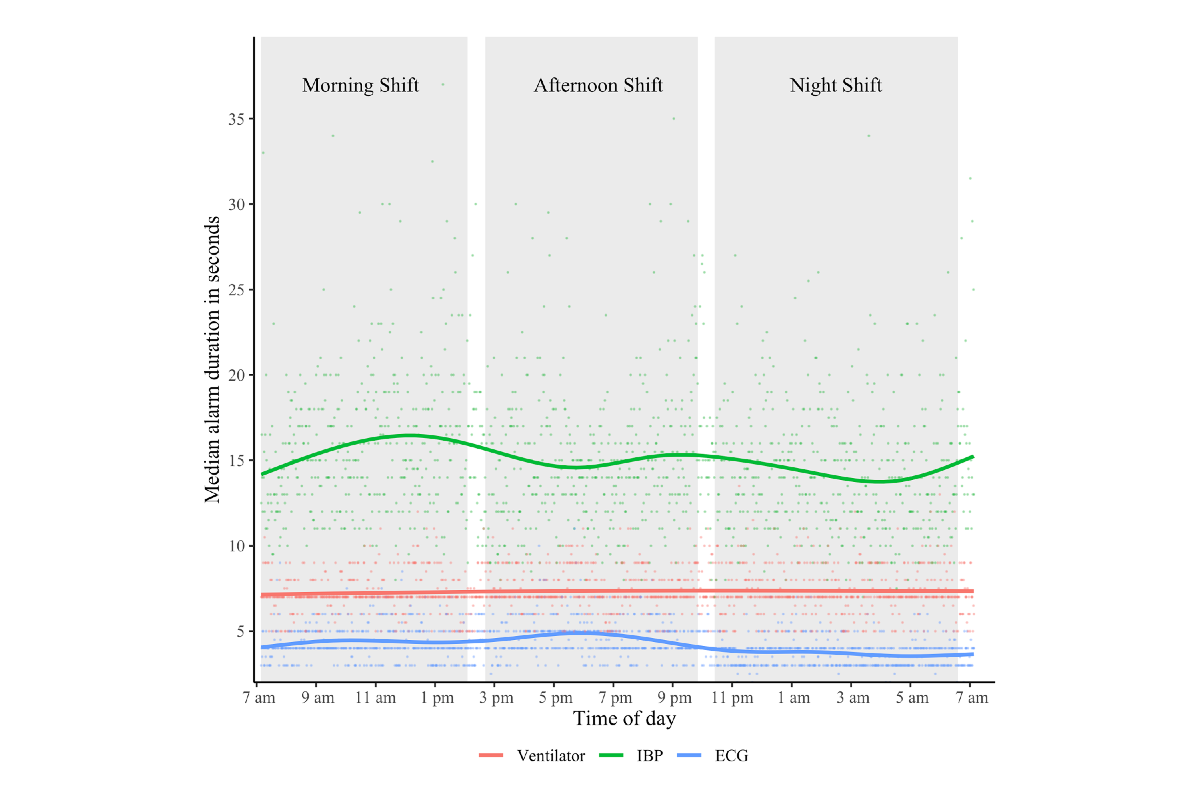
Median alarm duration of the 3 medical devices that issue most alarms over 24 hours. Each dot represents the median alarm duration for each minute of the day of the respective device. The line for each device is based on ggplot2’s smoothing function and represents a generalized additive model of the distribution (with the formula y ~ s(x, bs = "cs"). The white spaces between the grey bars (ie, shifts) visualize handover periods. ECG: electrocardiogram; IBP: invasive blood pressure.

**Figure 7:**
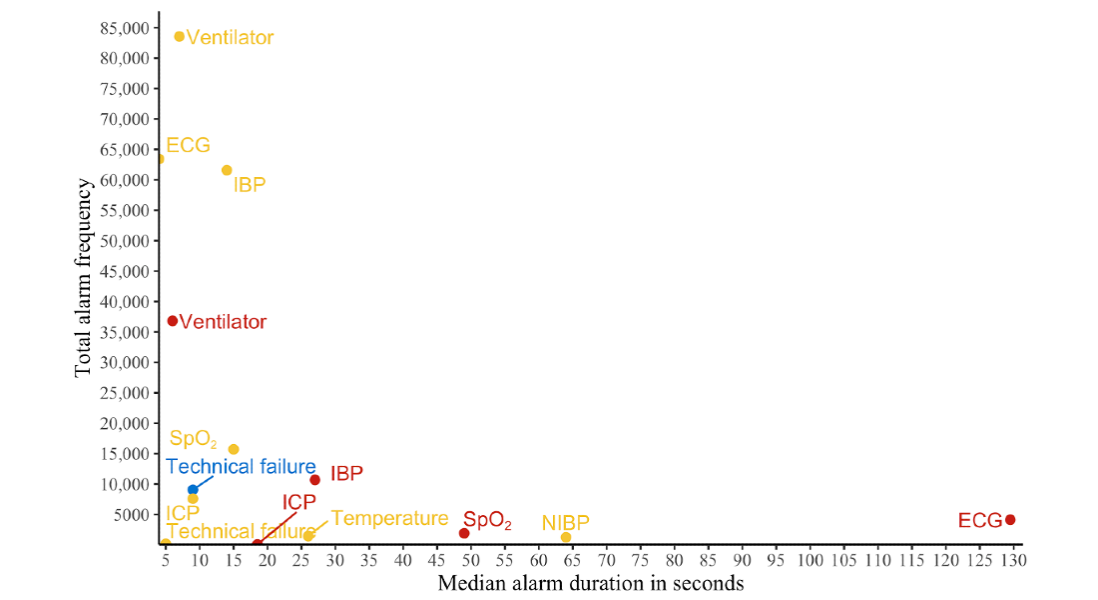
The median alarm duration from 8 medical devices plotted against the total number of alarms issued by the respective device. The colors correspond to the alarm criticalities (red, yellow, and blue). ECG: electrocardiogram; IBP: invasive blood pressure; ICP: intracranial pressure; NIBP: noninvasive blood pressure; SpO_2_: oxygen saturation.

Median alarm durations of yellow and red alarms were similar (8 seconds; range 0-22,048 seconds; range 0-28,531 seconds), while the median alarm duration of blue alarms was slightly longer (9 seconds; range 0-26,049 seconds). Regarding devices, the median duration of alarms by NIBP was the longest (64 seconds; range 0-3845 seconds), followed by temperature (26 seconds; range 0-7796 seconds), SpO_2_ (16 seconds; range 0-27,580 seonds), and IBP (14 seconds; range 0-28,531 seconds). ECG and the ventilator recorded the lowest median alarm duration (4 seconds; range 0-27,579 seconds and 7 seconds; range 0-22,048 seconds, respectively). Visualizing the alarm durations over 24 hours shows that the difference across devices was relatively stable over the course of an average day (see Figure 6). However, Figure 7 shows that there were substantial differences between the median duration to red and yellow ECG alarms.

#### Sensing

On average, the alarm pause function was applied 10.86 (SD 2.6) times per bed per day. Of all pauses that were started, 92% (14,719/16,002) were not actively terminated but lasted for their default maximum length of 3 minutes and therefore do not qualify as proper pauses [10]. The ICU’s proper pause-to-pause ratio is 0.09:1.

Of the ECG alarms, 16% (10,821/67,518) indicated a high respiratory rate, which amounted to 4% (10,821/297,830) of all alarms recorded in the data set, while the alarms from the ventilator related to a high respiratory rate covered another 11% (31,911/297,830) of all alarms. Additionally, both device groups had similar numbers of life-critical apnea alarms. This suggests an overmonitoring of the respiratory rate.

#### Exposure

A bed located in a single bedroom had, on average, 26% (172.9/137.2) more alarms per day than a bed located in a double bedroom. There were 32% more red alarms per bed in a single bedroom than in a double bedroom (2972.9/2250.3) and 25% more yellow alarms per bed in a single bedroom than in a double bedroom (12,638.8/10,105.2).

The calculated average alarms per bed per day yields 152.5 alarms (SD 42.2). On average, 36% (7.6/21, SD 1.6) of the 21 beds exceeded the units average every day, issuing on average 69% of all daily alarms (2199.9/3202.4, SD 651.2).

## Discussion

### Principal Findings

We aimed to provide technically versed ICU staff with a framework and the tools to conduct a self-analysis of patient monitoring alarm data in order to help them assess their unit’s alarm situation, inspect potential root causes of excessive alarms, and derive alarm management interventions that might help to remedy alarm fatigue. Our framework consists of 6 steps that should be iteratively applied: (1) define or re-evaluate metrics, (2) collect data, (3) analyze data, (4) visualize metrics, (5) present to staff and set goals together, and (6) design and implement interventions. We designed the framework to be useful independent of the ICU’s specialization (eg, COVID-19 units, neonatal ICUs, pediatric ICUs). In our observational study, we illustrated how the alarm log data of a large German ICU can be analyzed and how alarm metrics can be visualized using the scripts that we provide (steps 2 and 3 of the framework, respectively [20]). The data analysis was structured according to the aforementioned quality dimensions [12]: The alarm load was quantified by 152.5 (SD 42.2) alarms per bed per day on average, issued mostly by the ventilator, IBP measurement, and ECG in the morning shifts (ie, high/low blood pressure, high respiratory rate, high/low heart rate). Alarm flood conditions also mostly occurred in the morning shifts with, on average, 69.55 (SD 31.12) per day. With regard to avoidable alarms, technical alarms were mostly issued by the ECG (ie, lead fallen off). The dimension “responsiveness and alarm handling” included the metric “alarm duration.” The calculation yielded a median duration of 8 (range 0-2,291,314) seconds for clinical alarms and 7 (range 0-403,440) seconds for technical alarms. Regarding “sensing,” the alarm pause function is, on average, applied 10.86 (SD 2.6) times per bed per day, and in 92% (14,719/16,002) was not actively terminated, resulting in a proper pause-to-pause ratio of 0.09:1. The “exposure” to alarms per bed per day was higher in single rooms (26%, mean 172.9/137.2 alarms per day per bed). Most alarms were, on average, issued by 7.6 of 21 beds (36%).

### Alarm Metrics in Perspective

Cvach et al [29] suggested that most ICU patients have less than the average number of alarms per bed per day while a few have more than that. In their data analysis of adult telemetry, 19% (n=3) exceeded the unit’s average on a single day; in our data analysis, on average, 36% (n=7.6) exceeded the unit’s average.

Our data analysis shows that beds located in a single bedroom have a higher alarm load compared to a bed in a double bedroom. Further analysis of alarm data at the patient level could reveal whether the alarm load depends on the severity of the patient's illness. Upon presenting our results to ICU staff, they noted that patients in delirium are often treated in single bedrooms. Since delirium is a condition that can lead to erratic movements [31], this might explain the larger number of alarms coming from single bedrooms (eg, due to disconnected ECG leads). This anecdote highlights the importance of presenting the results of the data analysis to ICU staff, as suggested in Figure 1.

Slow response times can be an indicator of alarm fatigue [32]. However, response times to alarms can be slow for other reasons than alarm fatigue alone, such as the unit’s floor layout and policies [17] or the staff members’ individual personality traits [33]. Similarly, response times can be fast for other reasons than a well-functioning organization: ICU staff might be so severely desensitized to alarms that they start terminating them blindly, without properly evaluating the patient’s situation [12]. Although response time and alarm duration are related, they are not the same. Response time describes the time between the generation of an alarm and its manual termination. Alarm duration describes the time between the generation of an alarm and any termination of that alarm (such as the auto-termination of alarms if an alarm with a higher priority is issued). Our data did not include information on whether an alarm was manually terminated or not, which is why we focused on analyzing the alarm duration.

Although the pause function of the monitoring devices can prevent unnecessary alarms [14,26], it should be used responsibly by terminating it before leaving the patient. Our data yielded a proper pause-to-pause ratio of 0.09:1, meaning that most pauses that were started were not actively terminated but lasted for their default maximum length of 3 minutes. This ratio is far from Hüske-Kraus et al’s [12] ideal ratio of 1, where all pauses would be a “proper pause.” If it is indeed the case that health care providers leave patients with the alarm pause still engaged, then this aspect should be included in future staff training to promote a responsible use of the alarm pause function. However, other reasons for this pattern in the data should be considered as well. For example, sometimes even the maximum duration of an alarm pause is not long enough (eg, when ICU staff are in the middle of an intervention on the other side of the bed, unable to reach the patient monitor, watching the pause automatically disengage). Hence, this nonideal proper pause ratio does not necessarily represent carelessness of ICU staff but could hint towards the maximum default length of alarm pauses as being too short.

### Limitations

Quantification of the alarms does not reflect whether alarm fatigue is an issue in the respective ICU. In order to evaluate alarm fatigue as a complex sociotechnical phenomenon, the data analysis should be accompanied by a qualitative study (eg, by staff interviews or alarm fatigue surveys) [34]. Our applied grouping of metrics into dimensions is based on available literature, not delimited, and to some extent arbitrary. For example, the “alarm pause” metric could be assigned to the dimension “alarm handling” or the “alarm flood” metric to the dimension “exposure.” The software version of our monitoring system does not log technical alarms with a low priority (soft inoperable alarms), and we did not include alarms from every medical device that issues alarms (eg, perfusion pump alarms are not included). Hence, the metrics reported underestimate the actual alarm load of the unit.

### Conclusion

We demonstrated that basic data analysis skills can help generate valuable insights for designing alarm management interventions and how alarm data analyses might be embedded in an overarching framework that guides in developing such interventions. We hope the presented DIY instructions and the alarm processing and visualization scripts accompanying this publication will be helpful to other intensivists and researchers and spur the publication of many ICUs’ alarm data and lessons learned from their alarm management efforts.

## Appendix

Multimedia Appendix 1 Patient monitoring device group assignments.

Multimedia Appendix 2 Alarm notification abbreviations.

## Abbreviations

DIY: do-it-yourself
ECG: electrocardiogram
ICU: intensive care unit
IBP: invasive blood pressure
NIBP: noninvasive blood pressure
SpO_2_: oxygen saturation
